# Association between BDNF levels and suicidal behaviour: a systematic review protocol

**DOI:** 10.1186/s13643-015-0047-x

**Published:** 2015-04-24

**Authors:** Rebecca Eisen, Stefan Perera, Monica Bawor, Laura Banfield, Rebecca Anglin, Luciano Minuzzi, Zainab Samaan

**Affiliations:** MiNDS Neuroscience Graduate Program, McMaster University, 1280 Main Street West, Hamilton, ON L8S 4L8 Canada; Health Research Methodology Graduate Program, McMaster University, 1280 Main Street West, Hamilton, ON L8S 4L8 Canada; Department of Clinical Epidemiology and Biostatistics, McMaster University, 1280 Main Street West, Hamilton, ON L8S 4L8 Canada; Population Genomics Program, Chanchlani Research Centre, McMaster University, 1280 Main Street West, Hamilton, ON L8S 4L8 Canada; Health Sciences Library, McMaster University, 1280 Main Street West, Hamilton, ON L8S 4L8 Canada; Department of Psychiatry and Behavioural Neurosciences, McMaster University, 1280 Main Street West, Hamilton, ON L8S 4L8 Canada; Department of Medicine, McMaster University, 1280 Main Street West, Hamilton, ON L8S 4L8 Canada; Women’s Health Concerns Clinic, St. Joseph’s Healthcare Hamilton, 50 Charlton Avenue East, Hamilton, ON L8N 4A6 Canada; Peter Boris Centre for Addiction Research, St. Joseph’s Healthcare Hamilton, 100 West 5th Street, Hamilton, ON L8P 3R2 Canada

**Keywords:** Suicide, Attempted suicide, Suicidal ideation, Brain-derived neurotrophic factor, Systematic review, Protocol

## Abstract

**Background:**

Suicide is a worldwide public health concern that claims close to 1 million lives each year. Suicidal behaviour is a significant risk factor for completed suicide and is much more prevalent than completed suicide. Many internal and external factors contribute to the risk of suicidal behaviour. Recent research has focused on biological markers in suicide risk, including brain-derived neurotrophic factor (BDNF). BDNF is a protein involved in the growth, function, and maintenance of the nervous system. It has been implicated in psychiatric disorders and suicide. While some evidence suggests that reduced levels of BDNF are associated with suicide, the precise relationship has yet to be determined. The aim of this study is to review the literature examining the relationship between levels of BDNF and suicidal behaviour.

**Methods:**

A predefined search strategy will be implemented to search the following electronic databases: PubMed/MEDLINE, Excerpta Medica Database (EMBASE), PsycINFO, and Cumulative Index to Nursing and Allied Health Literature (CINAHL) from inception. The articles will be screened by two independent authors (RE and SP) using predetermined inclusion and exclusion criteria. Discrepancies will be resolved by consensus, or by a third author (ZS) in cases of disagreement. The primary outcome will be the association between levels of BDNF and suicidal behaviour. A meta-analysis will be conducted if appropriate. Quality of evidence and risk of bias will be evaluated.

**Discussion:**

The findings of this review will assist in identifying and treating individuals at increased risk of suicide.

**Systematic review registration:**

PROSPERO CRD42015015871.

**Electronic supplementary material:**

The online version of this article (doi:10.1186/s13643-015-0047-x) contains supplementary material, which is available to authorized users.

## Background

Suicide is a worldwide public health concern. It claims the lives of over 800,000 people each year, and the numbers continue to increase [1]. Suicide has a devastating impact at a number of levels, including the individual, family, community, and society. Suicidal behaviour encompasses a complex set of ideas, plans, and acts intended to end one’s own life. It occurs 10 to 20 times more often than completed suicide and is a significant risk factor for completed suicide in the general population [1,2].

While the causes of suicide are unclear, a number of internal (biological and psychological) and external (social and environmental) factors are thought to contribute to the risk of suicidal behaviour. Internal risk factors include psychiatric disorders (especially mood disorders), substance use disorders, chronic illness, and demographic variables (age and sex) [3]. External factors include unemployment, unmarried status, and a lack of social support [2,3]. While having a psychiatric disorder significantly increases one’s risk of suicide [4], and about 90% of people who attempt or complete suicide have a psychiatric disorder [3], most individuals with psychiatric disorders never attempt suicide. This indicates that there may be a predisposition toward suicidal behaviour independent of the underlying psychiatric disorders [2]. As well, many cases of suicide cannot be explained by the conventional risk factors proposed by clinical and research observations. Therefore, the focus has shifted to the investigation of biological markers in suicide risk, which has become more common among the recent literature.

The role of neurotrophins has been explored in relation to psychiatric disorders, including depression, bipolar disorder, anxiety, and schizophrenia [5]. Neurotrophins are a family of proteins that regulate the survival, development, maintenance, and function of vertebral nervous systems [6]. Brain-derived neurotrophic factor (BDNF) is the most abundant member of the neurotrophin family [6] and has been implicated in both suicide and suicidal behaviour [7]. BDNF is expressed in the brain and in other body tissues such as skeletal muscle and circulates throughout the body in the bloodstream [7,8]. When BDNF is released by a cell, it triggers a cascade of events that lead to neurogenesis, nerve growth, neuroplasticity, and neurotransmission [7]. BDNF is also important in morphological plasticity, neurite outgrowth, phenotypic maturation, and protein synthesis for neuron and synaptic functioning [6]. Since BDNF is intrinsic to these important processes, pathological changes in BDNF levels are likely involved in neurological deficits that impair one’s ability to adapt to difficult situations. Altered levels of BDNF may be responsible for the cognitive deficits and altered brain structure associated with depression, stress, and suicide.

Some studies have linked reduced levels of BDNF to psychiatric disorders and suicide. Low levels of serum BDNF are associated with a dispositional vulnerability to depression and with acute depressive states in the general population [9]. Lower levels of both serum and plasma BDNF are associated with major depressive disorder, and serum levels in particular have been correlated to severity of depression [10,11]. Stress, which plays an important role in suicidal behaviour and constitutes a major risk factor [7,12], is associated with altered levels of BDNF in the brain [13-16]. But while brain levels of BDNF are altered in depression and stress, evidence suggests a differential role of BDNF depending on the location in the brain. Depression and stress are associated with low levels of BDNF in the hippocampus and prefrontal cortex but high levels of BDNF in the amygdala and nucleus accumbens [17]. Antidepressants have been shown to normalize levels of BDNF expression [18].

Postmortem studies of the brains of suicide victims have revealed abnormally low levels of BDNF and its receptor, tropomyocin receptor kinase B (TrkB), compared to controls [19,20]. Interestingly, this was true regardless of the psychiatric diagnosis. Other studies have measured peripheral levels of BDNF in the blood of suicidal individuals. Deveci and colleagues compared serum BDNF levels among suicide attempters, depressed patients, and healthy controls [21]. Mean serum BDNF was significantly lower in both the suicidal group and the depressed group compared to the controls. Studies of plasma BDNF have found decreased levels in suicidal depressed patients compared with nonsuicidal depressed patients [22,23].

BDNF is a modifiable risk factor for suicidal behaviour. However, relatively few studies have investigated the relationship between levels of BDNF and suicidal behaviour. As well, since BDNF levels are altered in both depression and suicide, it is unclear whether the differences are related specifically to suicide. A systematic review is needed in order to summarize the existing studies and determine whether BDNF levels are in fact associated with suicidal behaviour, as well as to identify gaps in the literature that require further research.

### Objectives

The objective of this systematic review is to elucidate the association between levels of BDNF and suicidal behaviour (including completed suicide, attempted suicide, and suicidal ideation) in an adult population through a methodological summary of the literature.

The study goals are the following:

1. To investigate the relationship between levels of BDNF and suicidal behaviour by summarizing primary studies that have examined this relationship.
2. To combine the results of primary studies in a statistical manner using meta-analysis, when appropriate.
3. To critically evaluate the existing literature and identify which areas require additional research.

## Methods/Design

### Inclusion and exclusion criteria

This systematic review will include published observational studies (case–control and cohort studies) of central and peripheral levels of BDNF (including postmortem brain tissue, cerebrospinal fluid, plasma, serum, whole blood, and urine) and suicidal behaviours (including completed suicide, attempted suicide, and suicidal ideation) in a population aged 18 and older. Included studies will have investigated the association between levels of BDNF and suicidal behaviour by comparing BDNF levels between groups with and without suicidal behaviour. This review will include clinical samples as well as community-based samples. No demographic limitations will be applied apart from age, and no special populations will be excluded (e.g. incarcerated individuals, pregnant women, etc.).

### Search strategy

All relevant studies will be identified with no language or time restrictions. The databases to be searched from inception are as follows: PubMed/MEDLINE, PsycINFO, Excerpta Medica Database (EMBASE), and Cumulative Index to Nursing and Allied Health Literature (CINAHL). The search strategy (presented in Table 1) will use relevant keywords and their associated medical subject headings (MeSH). A number of different search terms related to suicidal behaviour that are common in the literature will be used in order to encompass this broad topic, including “suicide”, “attempted suicide”, “self-injurious behaviour”, “self harm*”, “automutilation”, “self inflict*”, and “suicidal ideation.” These terms will be combined with the term “brain-derived neurotrophic factor” or “BDNF”. The search will include titles, abstracts, and keyword fields. The reference lists from the included articles will be scanned manually to identify additional studies. The grey literature will be searched using the ProQuest Dissertations and Theses Database. Reviews, abstracts, and commentaries will be excluded. No language restrictions will be applied. An experienced health sciences librarian (LB) was consulted and assisted in the search strategy. A search alert will be set up to ensure the retrieval of relevant studies published after the initial search.

**Table 1:** **Search strategy for retrieval of relevant articles from multiple databases**

**Database**	**Search strategy**
MEDLINE (*n* = 106)	1. exp Suicide/
	2. suicid*.mp.
	3. exp Self-Injurious Behavior/
	4. (self harm* or self inflict* or self injur* or self wound* or self mutilat*).mp.
	5. automutilat*.mp
	6. 1 or 2 or 3 or 4 or 5
	7. brain derived neurotrophic factor.mp. or Brain-Derived Neurotrophic Factor/
	8. bdnf.mp
	9. 7 or 8
	10. 6 and 9
EMBASE (*n* = 366)	1. exp suicidal behaviour/
	2. suicid*.mp.
	3. exp automutilation/
	4. (self harm* or self inflict* or self wound* or self mutilat* or autmutilat*).mp.
	5. 1 or 2 or 3 or 4
	6. brain derived neurotrophic factor.mp. or exp brain derived neurotrophic factor/
	7. bdnf.mp.
	8. 6 or 7
	9. 5 and 8
PsycINFO (*n* = 76)	1. exp Suicide/
	2. exp Attempted Suicide/
	3. exp Suicidal Ideation/
	4. suicide*.mp
	5. exp Self Injurious Behavior/
	6. (self harm* self injur* or self inflict* or self wound* or self mutilat* or autmutilat*).mp.
	7. 1 or 2 or 3 or 4 or 5 or 6
	8. brain derived neurotrophic factor.mp. or exp Brain Derived Neurotrophic Factor/
	9. bdnf.mp.
	10. 8 or 9
	11. 7 and 10
CINAHL (*n* = 4)	1. MH (“Suicide+”)
	2. “suicid*”
	3. MH (“Self-Injurious Behavior”)
	4. “self harm*” OR “self injur*” OR “self inflict*” OR “self wound*” OR “self mutilat*” OR “automutil*”
	5. 1 or 2 or 3 or 4
	6. “brain derived neurotrophic factor”
	7. “bdnf”
	8. 6 or 7
	9. 5 and 8

CINAHL, Cumulative Index to Nursing and Allied Health Literature; EMBASE, Excerpta Medica Database.

### Data screening

All citations and abstracts retrieved using the predefined search strategy (Table 1) will be screened by two raters (RE and SP) independently. Eligible articles will be identified using pre-established criteria and retrieved for full-text review. Disagreements at any point in the review process will be resolved by discussion. In cases where consensus is not reached, eligibility will be determined by a third author (ZS). Studies that are ineligible will be excluded from review. The reasons for exclusion will be recorded and described in the flow diagram (see Additional file 1: Figure S1). For each phase of screening, the Kappa statistic will be used to calculate inter-rater agreement [24]. The authors of the studies will be contacted for clarification and additional data when necessary.

### Data extraction

The two raters (RE and SP) will extract data independently from the included studies using a pre-established data extraction form that will be pilot tested beforehand (see Additional file 2). The raters will obtain the following information from each study: first author, year of publication, city and country of publication, article title, journal, study design, description of sample population, mean age, ethnicity, and definition of suicidal behaviour. For studies that include more than one measure of suicidal behaviour, each measure will be recorded. This will allow for the combination of studies with the same measures in a meta-analysis. For example, some studies have used the suicide item in Hamilton Depression Rating Scale (HDRS), while others have used the Beck Scale for Suicidal Ideation (BSS) [25,26]. We will record all measures reported so that we can combine, for instance, all studies that used the BSS. We expect most studies to report suicidal behaviour as a dichotomous measure (e.g. history of suicide attempt or no history of suicide attempt). However, if studies used different measurement scales to indicate severity of suicidal behaviour, then we will use a dichotomized outcome based on the presence of suicidal behaviour, regardless of severity. For studies that report multiple time points for suicidal behaviour (e.g. suicide attempt within the last month vs. the past 3 months vs. lifetime), all time points will be recorded. This will allow for the combination of similar time frames when possible. Information regarding the BDNF measurements will be obtained, including the tissue sample in which it was measured, the lab analysis methods, the mean BDNF levels and standard deviations, and the unit of measurement used. If relevant, the comparison group and any underlying psychiatric disorders will also be recorded. For each study, primary and secondary outcome measures, results, statistical analyses, and conclusions will be recorded. If any data are missing or incomplete, authors will be contacted for additional details.

### Assessment of quality

The risk of bias of included studies will be assessed by two independent raters (RE and SP) using the Newcastle-Ottawa Scale (NOS) [27]. An adapted version of the NOS will be used, in keeping with previous systematic reviews of observational studies (see Additional file 3) [28]. This version of the NOS contains seven questions in the following domains: methods for selecting study participants (selection bias), methods to control for confounding (performance bias), statistical methods (detection bias), and methods of exposure and outcome assessment (information bias). Risk of bias will be assessed on a scale from 0 to 3, where 0 indicates high risk of bias and 3 indicates low risk. Descriptions and examples of high and low risk of bias are provided. This adapted NOS also includes categories related to statistical methods, confounding effects, and reporting of missing data. The Grading of Recommendations, Assessment, and Evaluation (GRADE) framework will be used to report the quality of evidence and strength of recommendations [29]. This framework provides a systematic approach for considering and reporting risk of bias, imprecision, inconsistency, indirectness of study results, and publication bias. A summary of findings table will be presented to allow for assessment of confidence in the estimates.

### Statistical analyses and heterogeneity

The results of this systematic review will be presented as a qualitative summary of the literature. When possible, meta-analyses will be performed. This review will encompass a wide variety of studies with different designs, sample populations, BDNF measurements, and definitions of suicidal behaviour. Clinical and methodological heterogeneity are expected. Therefore, separate meta-analyses will be conducted on groups of studies that share the following characteristics:

1. Study design (e.g. case control vs. cohort)
2. Definition of suicidal behaviour (completed suicide, attempted suicide, or suicidal ideation)
3. Type of tissue from which BDNF was sampled (e.g. plasma, serum, brain tissue)

Meta-analyses will be performed using the extracted data from groups of studies if the following conditions are met:

- More than one study is found that share all of the characteristics listed above
- There are minimal differences among the studies in other relevant characteristics (such as sample population)
- Data in each study are available and reported with sufficient detail.

Heterogeneity will be assessed using the *I*^2^ statistic. The interpretation of the I^2^ value will be based on the guidelines in the Cochrane Handbook for Systematic Reviews of Interventions, which defines 0% to 40% as low heterogeneity, 30% to 60% as moderate heterogeneity, 50% to 90% as substantial heterogeneity, and 75% to 100% as considerable heterogeneity [30]. In this study, an *I*^2^ value below 50% will be considered low heterogeneity. The *P* value from the chi-squared test will also be taken into consideration, with significant heterogeneity being defined with a *P* value below 0.10. Groups of studies in which heterogeneity is found to be low (*I*^2^ < 50%) will be assessed in a combined statistical manner using meta-analysis. The mean differences (MD) in the BDNF level between groups with and without suicidal behaviour will be combined into a summary estimate. Only adjusted values extracted from the primary studies will be used. A random-effects model will be implemented, as it accounts for both within-study and between-study variability. As well, a mixed-effects model will be used to examine the possible mediation effect of BDNF on the relationship between other variables (including sex, age, and psychiatric diagnosis) and risk of suicidal behaviour. Sensitivity analysis will be conducted based on risk of bias; studies with a score of 0 on the NOS will be excluded to determine whether the summary estimate stays the same.

The main source of heterogeneity hypothesized is clinical heterogeneity, resulting from diversity in the populations being studied. Some studies have derived their samples from particular clinical populations, such as depressed patients, while others have sampled populations with a range of psychiatric diagnoses, or community-based populations. Since alterations in BDNF levels are linked to psychiatric disorders, particularly depression, the sample characteristics could have a significant influence on the associations between BDNF levels and suicidal behaviour [5,9]. The implications of this heterogeneity on the interpretation of the results will be discussed.

In the event that the heterogeneity is too high to allow for meta-analyses to be performed, the results of this systematic review will be presented as a narrative summary of the literature examining the relationship between levels of BDNF and suicidal behaviour. The included studies will be synthesized in a comprehensive, up-to-date review of this emerging area of research.

### Presenting and reporting of results

This systematic review will be performed and presented according to the Preferred Reporting Items for Systematic Reviews and Meta-Analyses (PRISMA) guidelines, as well as the Meta-analysis of Observational Studies in Epidemiology (MOOSE) guidelines [31,32]. The article selection process will be summarized in a flow diagram (see Additional file 1: Figure S1). The relevant outcomes and characteristics of each study will be reported in summary tables. Publication bias will be assessed using Egger’s plot.

This protocol follows the Preferred Reporting Items for Systematic Review and Meta-Analysis Protocols (PRISMA-P) 2015 Statement [33].

## Discussion

This systematic review will present evidence from which conclusions can be made regarding the relationship between levels of BDNF and suicidal behaviour. It is expected that an inverse correlation will be found, with reduced levels of BDNF associated with suicidal behaviour. The findings of this systematic review will contribute to our understanding of BDNF as a biological factor involved in suicide risk, and of suicide pathology more generally. These findings, as well as the appraisal of the status of the literature, will be of use to clinicians, in identifying individuals at increased risk of suicide, and researchers, in developing therapeutic targets.

## Additional files

Additional file 1: Figure S1. PRISMA flow diagram. Additional file 2:
**Data extraction form.**
Additional file 3:
**Modified NOS.**

## Abbreviations

BDNF: brain-derived neurotrophic factor
TrkB: tropomyocin receptor kinase B
NOS: Newcastle-Ottawa Scale
