# Supplementary figures and images for: Association between BDNF levels and suicidal behaviour: a systematic review protocol
Source: Syst Rev. 2015 Apr 24;4:56. doi: 10.1186/s13643-015-0047-x (PMC4410596; doi:10.1186/s13643-015-0047-x)

Figure 1: PRISMA Flow Diagram

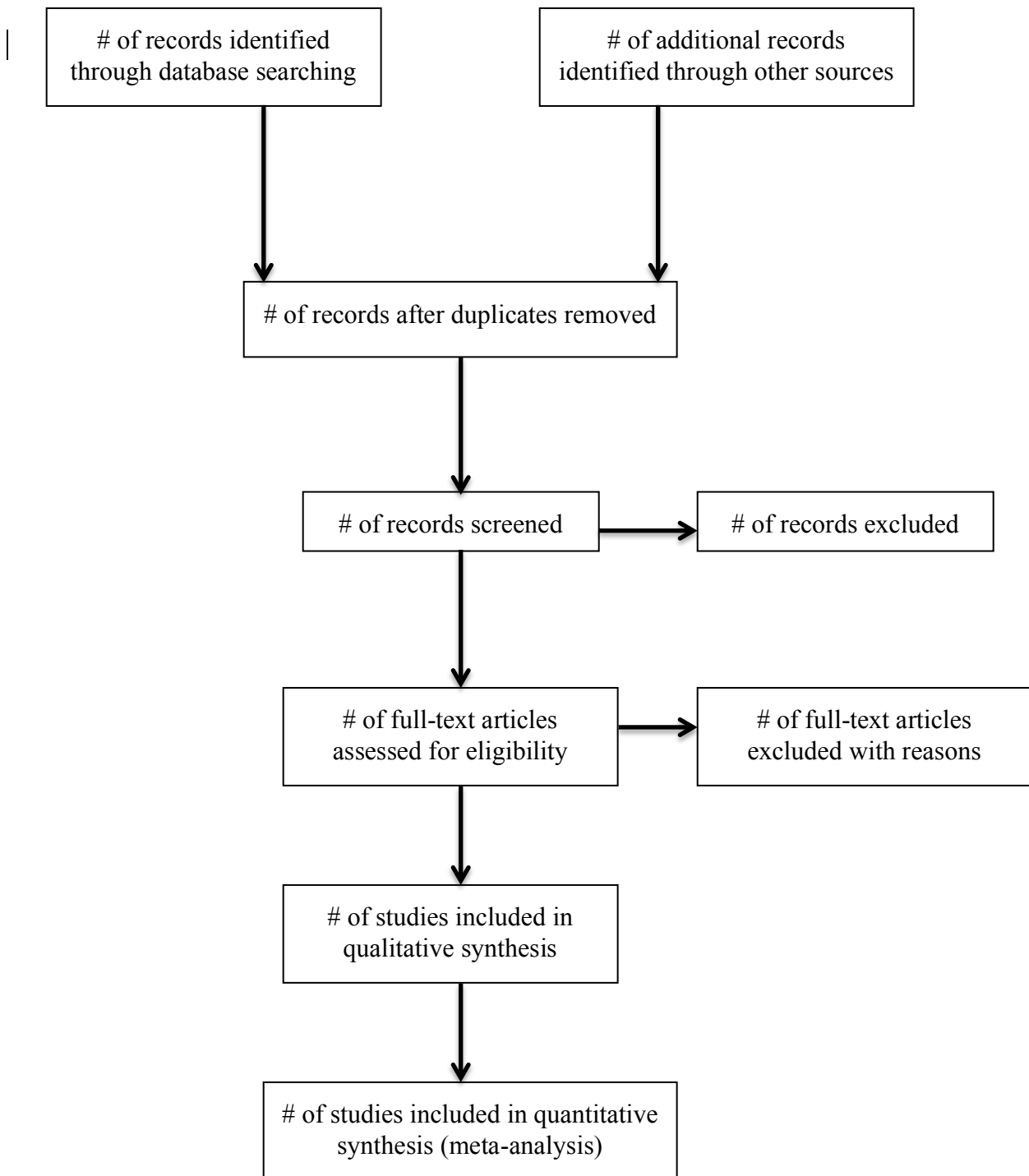

Supplement: Additional file 1: Figure S1. — PRISMA flow diagram. [file 13643_2015_47_MOESM1_ESM.pdf]
