# Supplementary material for: Association between BDNF levels and suicidal behaviour: a systematic review protocol
Source: Syst Rev. 2015 Apr 24;4:56. doi: 10.1186/s13643-015-0047-x (PMC4410596; doi:10.1186/s13643-015-0047-x)
Supplement: Additional file 2: — Data extraction form. [file 13643_2015_47_MOESM2_ESM.pdf]

<u>**Data Extraction Form**</u>

Association Between BDNF Levels and Suicidal Behaviour: A Systematic Review

Study ID: ______ Reviewer Initials: _______

<u>STUDY INFORMATION</u>

Last Name of First Author, Initial: ____________________ Year of Publication__________

Title of Article: ____

Journal Name: _______________________________ City, Country: ____________________

<u>METHODS</u>

Study Setting: _____________________________ Study Design: _______________________

Description of sample and comparison groups:

____

Sample Size: Total________, Men _________, Women _________

Per group ____

Mean Age (SD): Total ______, Men _________, Women _________

Per group ____

Ethnicity: _____________________________________

<u>RESULTS</u>

Definition of suicidal behaviour: ____

Type of BDNF sample: ______________________Lab analysis method: ___________________

Mean (SD) and unit of measurement of BDNF in each group:

____

Outcome measures: ____

Number of individuals experiencing the event: Completed suicide ________,

Attempted suicide ________, Suicidal ideation _________, Other _________

Statistical Methods: ____

Adjusted for: ____

Statistical Results: Coefficient ______________, 95% CI ____________ p-value _____________

Findings: ____

Limitations: ____

INCLUSION CRITERIA

The study examines the association between BDNF levels and risk of suicidal behaviour
Study participants are ≥ 18 years of age
Study participants are human
Study has been completed and published

EXCLUSION CRITERIA

Includes children or adolescents
Animal study
Incomplete study
Abstract, commentary, or review

COMMENTS

____

____
